# Supplementary material for: Sarcopenia is not associated with inspiratory muscle strength but with expiratory muscle strength among older adults requiring long-term care/support
Source: PeerJ. 2022 Feb 17;10:e12958. doi: 10.7717/peerj.12958 (PMC8858577; doi:10.7717/peerj.12958)
Supplement: Supplemental Information 1 — CI, confidence interval; MEP, maximal expiratory pressure; FEV1.0%, forced expiratory volume % in one second. Dependent variables: Non-sarcopenia = 0, sarcopenia = 1. Independent variables: Respiratory parameters (forced vital capacity, FEV1.0%; peak expiratory flow rate; MEP, maximal inspiratory pressure) were considered as independent variables by a stepwise method. Age was used as a control variable. [file peerj-10-12958-s001.docx]

**Table S1. Association between sarcopenia and respiratory parameters based on binomial logistic regression analysis by sex**

|  | β | P-value | Odds ratio | 95%CI |
| --- | --- | --- | --- | --- |
| Men |  |  |  |  |
| MEP (cmH_2_O) | -0.033 | 0.022 | 0.968 | 0.941–0.995 |
| Age (years) | 0.062 | 0.097 | 1.064 | 0.989–1.145 |
| Women |  |  |  |  |
| MEP (cmH_2_O) | -0.070 | 0.006 | 0.933 | 0.887–0.980 |
| FEV1.0% (%) | 0.087 | 0.068 | 1.091 | 0.993–1.199 |
| Age (years) | -0.046 | 0.206 | 0.955 | 0.889–1.026 |

CI, confidence interval; MEP, maximal expiratory pressure; FEV1.0%, forced expiratory volume % in one second.

Dependent variables: Non-sarcopenia=0, sarcopenia=1.

Independent variables: Respiratory parameters (forced vital capacity, FEV1.0%, peak expiratory flow rate, MEP, maximal inspiratory pressure) were considered as independent variables by a stepwise method. Age was used as a control variable.
