# Supplementary material for: Sarcopenia is not associated with inspiratory muscle strength but with expiratory muscle strength among older adults requiring long-term care/support
Source: PeerJ. 2022 Feb 17;10:e12958. doi: 10.7717/peerj.12958 (PMC8858577; doi:10.7717/peerj.12958)
Supplement: Supplemental Information 2 — BMI, body mass index; SMI, skeletal muscle mass index; MEP, maximal expiratory pressure; MIP, maximal inspiratory pressure. [file peerj-10-12958-s002.docx]

**Table S2. Pearson's correlation of MEP and MIP with component factors by sex**

|  | Men | | | | Women | | | |
| --- | --- | --- | --- | --- | --- | --- | --- | --- |
|  | MEP | P-value | MIP | P-value | MEP | P-value | MIP | P-value |
| Height (cm) | 0.144 | 0.243 | 0.058 | 0.642 | 0.165 | 0.220 | 0.044 | 0.747 |
| Weight (kg) | 0.396 | 0.001 | 0.183 | 0.137 | 0.422 | 0.001 | 0.301 | 0.023 |
| BMI (kg/m^2^) | 0.328 | 0.007 | 0.153 | 0.216 | 0.377 | 0.004 | 0.300 | 0.023 |
| Grip strength (kg) | 0.297 | 0.015 | 0.108 | 0.386 | 0.291 | 0.028 | 0.132 | 0.329 |
| Usual gait (m/s) | 0.196 | 0.111 | 0.145 | 0.243 | 0.326 | 0.013 | 0.100 | 0.461 |
| SMI (kg/m^2^) | 0.264 | 0.031 | 0.066 | 0.597 | 0.336 | 0.011 | 0.126 | 0.349 |
| Trunk muscle mass per  height squared (kg/m^2^) | 0.327 | 0.007 | 0.082 | 0.511 | 0.361 | 0.006 | 0.215 | 0.108 |
| MEP (cmH_2_O) | — | — | 0.630 | <0.001 | — | — | 0.388 | 0.003 |
| MIP (cmH_2_O) | 0.630 | <0.001 | — | — | 0.388 | 0.003 | — | — |

BMI, body mass index; SMI, skeletal muscle mass index; MEP, maximal expiratory pressure; MIP, maximal inspiratory pressure.
